# HPV18 E1^E4 is assembled into aggresome-like compartment and involved in sequestration of viral oncoproteins.

**DOI:** 10.3389/fmicb.2013.00251

**Published:** 2013-08-27

**Authors:** Naoko Kajitani, Ayano Satsuka, Satoshi Yoshida, Hiroyuki Sakai

**Affiliations:** ^1^Laboratory of Mammalian Molecular Biology, Department of Molecular and Cellular Biology, Graduate School of Biostudies, Kyoto UniversityKyoto, Japan; ^2^Laboratory of Gene Analysis, Department of Viral Oncology, Institute for Virus Research, Kyoto UniversityKyoto, Japan; ^3^Department of Viral Oncology, Graduate School of Medicine, Kyoto UniversityKyoto, Japan

**Keywords:** HPV, E1^E4, aggresome, HPV oncoproteins, HPV replication

## Abstract

Papillomavirus is the etiological agent for warts and several squamous carcinomas. Skin cancer induced by cottontail rabbit papillomavirus was the first animal model for virus-induced carcinogenesis. The target organ of the virus infection is stratified epithelium and virus replication is tightly regulated by the differentiation program of the host cell. E1^E4 protein is a viral gene product, and although it is considered to be involved in the control of virus replication, little is known about the biological role. We found that HPV18 E1^E4 was assembled into an aggresome-like compartment and was involved in sequestration of virus oncoproteins, which might contribute to the differentiation-dependent lifecycle of papillomavirus.

## INTRODUCTION

Papillomavirus is a small virus containing a double-stranded circular DNA as its genome (zur Hausen, 2002). Genomic DNA of typical papillomavirus, human papillomavirus type 16 (HPV16) or HPV18 is ca. 8 kb long and coding six regulatory genes (E1, E2, E4, E5, E6, E7) and two structural genes (L1, L2). Papillomaviruses are found in almost all mammals and also in amniotes. The virus infects to stratified epithelium organ, such as cutaneous or mucosal membrane, and the infection causes various types of hyperplasia. It is known that the infections of some types of papillomaviruses occasionally induce malignant tumors. The cancer formation by the infection of cottontail rabbit papillomavirus (CRPV) was the first animal model of virus-induced carcinogenesis (Campo, 2002).

The replication of papillomavirus is regulated by the differentiation program of the host cell (Doorbar, 2005). The target cell of the virus infection is basal cell of stratified epithelium, in which the virus replication maintains latent status. Cell division of the infected basal cell produces a daughter cell, and the daughter cell is moved to the surface region of the epithelium and proceeds to differentiate. Virus gene expression and genome replication are enhanced in accordance with the cell differentiation, and the productive replication occurs in fully differentiated cells (Sakakibara et al., 2013). The regulatory mechanism of the differentiation-dependent viral replication remains largely unknown.

A variety of mRNAs are produced by alternative splicing in HPV (Schwartz, 2013). About E4 gene, 5′ region of E1 is jointed to E4 coding sequence by RNA splicing, then the gene product contains five amino acid residues of E1 at the N-terminus of the protein coded by E4 ORF, which is called “E1^E4”. By the analysis of the specimens obtained from infected individuals and animals, the expression level of E1^E4 appeared to be intense in differentiated layers of the infected lesions (Sterling et al., 1993; Doorbar et al., 1997), suggesting that E1^E4 is involved in the productive stage of viral replication. It was reported on CRPV that the E1^E4 was required for the viral DNA amplification and the late protein expressions (Peh et al., 2004). E1^E4s of HPV16 and HPV31 were reported to be involved in viral genome amplification and cell cycle maintenance in S-phase of differentiated cells (Nakahara et al., 2005; Wilson et al., 2005). HPV16 E1^E4 was also reported to be required for viral genome maintenance in undifferentiated basal cells (Nakahara et al., 2005). There was a paper describing that HPV18 E1^E4 was participated in viral genome amplification and the late gene expression in differentiated cells, although it was not involved in the viral genome maintenance or the S-phase maintenance of differentiated cells (Wilson et al., 2007). With these findings, E1^E4 could be considered to play a role in productive phase of virus replication.

Several biological and biochemical properties of E1^E4 were reported previously. HPV16 E1^E4 interacts with cytokeratins and collapses the cytokeratin networks spreading in the cytoplasm (Doorbar et al., 1991). Phosphorylation of HPV16 E1^E4 by extracellular signal-regulated kinase (ERK) was reported to cause conformational change of E1^E4 and promote the interaction with cytokeratins (Wang et al., 2009).

The expression of E1^E4 of HPV16 orHPV18 induces G2/M cell cycle arrest (Davy et al., 2002; Nakahara et al., 2002) and the interaction between the E1^E4 and Cyclin A/B has been proposed to be involved in the cell cycle arrest (Davy et al., 2005, 2006). HPV16 E1^E4 was also reported to be involved in RNA processing through its association with E4-DEAD box protein (E4-DBP), a putative RNA helicase (Doorbar et al., 2000), in RNA metabolism (Bell et al., 2007), and in mitochondrial function (Raj et al., 2004). There was a report that HPV1 E4 induced the redistribution of nuclear domain 10 (ND10) body, which is a candidate site of the HPV genome replication (Roberts et al., 2003). These biological properties of E1^E4 might be involved in the HPV lifecycle, however, their precise roles in virus replication remain to be elucidated.

There is a self-association motif in the C-terminal region of E1^E4, and E1^E4s form aggregates in the cytoplasm through the motifs (Bryan et al., 1998). It was reported that the aggregate had amyloid-like structure (McIntosh et al., 2008). Several viruses were reported to utilize cytoplasmic aggregates called as “aggresome” for their replication (Wileman, 2007). Although the biological significance of the aggregate formed by E1^E4 was unknown, it might contribute to HPV lifecycle.

“Aggresome” was originally defined as a cytoplasmic compartment in which misfolded proteins are assembled (Johnston et al., 1998). Accumulation of misfolded proteins is toxic for cell viability as in the cases of neurological disorders including Parkinson’s, Alzheimer’s, and Huntchington’s diseases. To counteract the toxicity, misfolded proteins are refolded into native structure or eliminated by molecular chaperones or proteasomes, respectively. However, aggregated proteins exhibit resistance to proteolysis. The aggregates are assembled at microtubule organizing center (MTOC) region and form “aggresome”, for which the dynein-dependent retrograde transport along microtubules is involved. Aggresomes contain polyubiquitinated proteins, molecular chaperones, and histone deacetylase 6 (HDAC6), and are wrapped in vimentin cage. It is considered that aggresomes activate autophagy pathway and they are processed in autophagy-dependent manner (Kopito, 2000).

In order to investigate E1^E4 function, we searched for cellular factors that interact with 18E1^E4 protein, and vimentin was identified as a candidate. We also found the 18E1^E4 aggregates were wrapped with vimentin as “aggresomes.” In this report, we present the structure of 18E1^E4 aggregate and its possible role in HPV replication.

## MATERIALS AND METHODS

### CELL CULTURE, TRANSFECTION

HeLa, CV1 and 293T cells were maintained with Dulbecco’s modified minimal essential medium (DMEM) supplemented with 10% fetal bovine serum. The cells were transfected with plasmid DNA (5 μg) and herring sperm DNA (5 μg; Roche Diagnostics, GmbH, Mannheim, Germany) by a standard calcium phosphate coprecipitation method.

### DNA CONSTRUCTION

HPV18 and HPV11 genomic DNAs were provided by Dr. Peter M. Howley (Harvard Medical School, Boston, USA). 18E1^E4, 11E1^E4, 18E5, 18E6, and 18E7 cDNAs were obtained by a polymerase chain reaction (PCR). 18E1^E4 and 11E1^E4 cDNAs were cloned into pPC86 vector (Invitrogen^TM^, Life Technologies, Corp., Carlsbad, CA, USA), pGEX-5X (Promega Corp., Madison, WI, USA), pCMV_4_ (Nakahara et al., 2002), and pEGFP-C1 (Clontech Laboratories, Inc., Mountain View, CA, USA). 18E5, 18E6, and 18E7 cDNAs were cloned into pCMV7.1 (Sigma-Aldrich Corp., St. Louis, MO, USA) in order to express 3xFLAG-tagged proteins.

### YEAST TWO-HYBRID SYSTEM

We used ProQuest^TM^ Two-Hybrid System (Invitrogen^TM^, Life Technologies, Corp, Carlsbad, CA, USA). 18E1^E4 cDNA was cloned into pPC86 vector. For cDNA library, we used ProQuest^TM^ Human Fetal Brain cDNA Library (Invitrogen^TM^, Life Technologies, Corp., Carlsbad, CA, USA). Screening was performed by following manufacturer’s instruction.

### GST PULL DOWN ASSAY

Glutathione *S*-transferase (GST)-tagged 18E1^E4 and 11E1^E4 were expressed by using pGEX-5X vector (Promega Corp., Madison, WI, USA). The fusion proteins were expressed in *E. coli* (BL21 strain), and purified with Glutathione Sepharose 4B beads (GE Healthcare UK Ltd, Little Chalfont, Buckinghamshire, UK). ^35^S-methionine labeled protein was synthesized with TNT Quick Coupled Transcription/Translation Systems (Promega Corp., Madison, WI, USA). Vimentin cDNA was obtained by PrimeScript II 1st strand cDNA Synthesis Kit (Takara Bio Inc., Shiga, Japan) with mRNAs obtained from HeLa cells. The cDNA was cloned into pGEM-3Zf(+) (Promega Corp., Madison, WI, USA) for *in vitro* transcription/translation.

Purified GST-fusion proteins and ^35^S-Met labeled vimentin were incubated in a binding buffer [20 mM Tris–HCl (pH 7.5), 50 mM NaCl, 4 mM MgCl_2_, 0.5% Nonidet P-40, 2% skim milk, 2 mM dithiothreitol (DTT)] at 4°C for 2 h. The complex was subjected to sodium dodecyl sulfate (SDS)-polyacrylamide gel electrophoresis (SDS-PAGE), and the vimentin bound to GST-fusion protein was detected with BAS5000 (FUJIFILM Corp., Tokyo, Japan).

### IMMUNOPRECIPITATION AND IMMUNOBLOT

Total cell lysates were prepared with triple detergent lysis buffer [150 mM NaCl, 50 mM Tris–HCl (pH 8.0), 0.1% SDS, 1% Nonidet P-40, 0.5% sodium deoxycholate] supplemented with protease inhibitor cocktail (Nacalai Tesque, Kyoto, Japan) and 1 mM DTT. The cell lysates were centrifuged at 14,000 rpm for 10 min at 4°C, and the supernatants were used for immunoprecipitation and immunoblot. The supernatants were used as soluble fractions in several experiments. The pellets were resuspended in 2× SDS sample buffer [0.125 M Tris–HCl (pH6.8), 4% SDS, 0.2 M DTT, 20% glycerol, 0.001% bromophenol blue] and used as insoluble fractions. In our experiment, 10 μg of protein could be obtained from ca. 1 × 10^4^ cells as soluble fraction. For immunoblot analysis, 10 μg of soluble fraction was loaded into each lane. It was not feasible to measure the protein concentration of insoluble fraction, therefore the portion equivalent to 1 × 10^4^ cells was loaded into each lane.

For immunoprecipitation, the cell lysates, Protein-G agarose (Invitrogen Corp., Carlsbad, CA, USA) and an appropriate antibody were incubated in NET-Gel Buffer [150 mM NaCl, 50 mM Tris–HCl (pH7.5), 0.1% Nonidet P-40, 1 mM EDTA, 0.25% gelatin] at 4°C for ≥ 4 h. The complex bound to Protein-G agarose beads was washed six times, and then suspended in 6× SDS sample buffer [0.35 M Tris–HCl (pH6.8), 10% SDS, 0.6 M DTT, 30% glycerol, 0.012% bromophenol blue].

The immunoprecipitation samples or the cell lysates were subjected to SDS-PAGE, and blotted to a polyvinylidene difluoride (PVDF) membrane (Hybond-P; GE Healthcare UK Ltd, Little Chalfont, Buckinghamshire, UK). The immunoblot with anti-β-actin antibody (Clone AC-15; Sigma-Aldrich Corp., St. Louis, MO, USA) was used for checking the protein amount loaded on the gel. Following antibodies were used for immunoblot and immunofluorescence analyses; anti-FLAG polyclonal antibody (F7425), anti-FLAG monoclonal antibody (F3165; Sigma-Aldrich Corp., St. Louis, MO, USA), anti-vimentin antibody (sc-6260), anti-DnaJB6 (Hsp40) antibody (sc-100710), anti-HDAC6 antibody (sc-11420; Santa Cruz Biotechnology, Inc., Dallas, TX, USA), anti-γ-tubulin antibody (ab11316), anti-ubiquitin antibody (ab7780; Abcam plc., Cambridge, UK), and anti-p62 antibody (PM045; Medical & Biological Laboratory Co., Ltd, Nagoya, Japan). Horseradish peroxidase (HRP)-conjugated secondary antibodies and a luminal reagent (ECL-prime) were purchased commercially (GE Healthcare UK Ltd, Little Chalfont, Buckinghamshire, UK). The chemiluminescent signal was visualized with a chemiluminescent image analyzer (LAS-3000; FUJIFILM Corp., Tokyo, Japan).

### IMMUNOFLUORESCENCE ANALYSIS

For IFA, the cells on cover glasses were fixed with 4% paraformaldehyde (PFA) at room temperature for 5 min or cold methanol (for γ-tubulin staining) at -20°C for 20 min, permeabilized with 0.1% Nonidet P-40/phosphate buffered saline (PBS) followed by blocking with 5% non-fat dry milk. The samples were incubated with each primary antibodies diluted as manufacturer’s instruction. Alexa Fluor^®^ 488 or 546 labeled secondary antibodies were purchased commercially (Molecular Probes^®^, Life Technologies Corp., Carlsbad, CA, USA). Fluorescence microscope (Axiovert200 and AxioVision; Carl Zeiss Microscopy GmbH, Jena, Germany) and confocal laser microscope (TCS SP2 AOBS, Leica Microsystems GmbH, Wetzlar, Germany) were used for analysis.

### CHEMICAL INHIBITORS

At 24 h after transfection, chemical inhibitors were added into the culture medium. After incubation for 24 h, the cells were harvested to obtain cell lysates, or fixed for IFA. Nocodazole (Sigma-Aldrich Co., St. Louis, MO, USA), MG132 (Wako Pure Chemicals Industries, Ltd, Osaka, Japan), ciliobrevin D (Merck KGaA, Darmstadt, Germany), and tubacin (Santa Cruz Biotechnologies, Inc., Dallas, TX, USA) were purchased commercially, solubilized in DMSO, and used at 10, 10, 20, and 10 μM, respectively, as working concentration.

## RESULTS

### INTERACTION BETWEEN HPV18 E1^E4 AND VIMENTIN PROTEINS

To investigate the biological function of HPV E1^E4, we searched for cellular factors that interact with HPV18 E1^E4 protein (18E1^E4). For screening, we used the yeast two-hybrid assay with 18E1^E4 as the bait. Among several factors identified from screening, we focused on vimentin, a cytoskeletal protein categorized as a type III intermediate filament. It is known that vimentin is involved in various cellular events, including cell division and signal transduction (Ivaska et al., 2007); therefore, we considered that the interaction between 18E1^E4 and vimentin might induce a modification of the cellular structure or function to adapt it in favor of virus replication.

**FIGURE 1:**
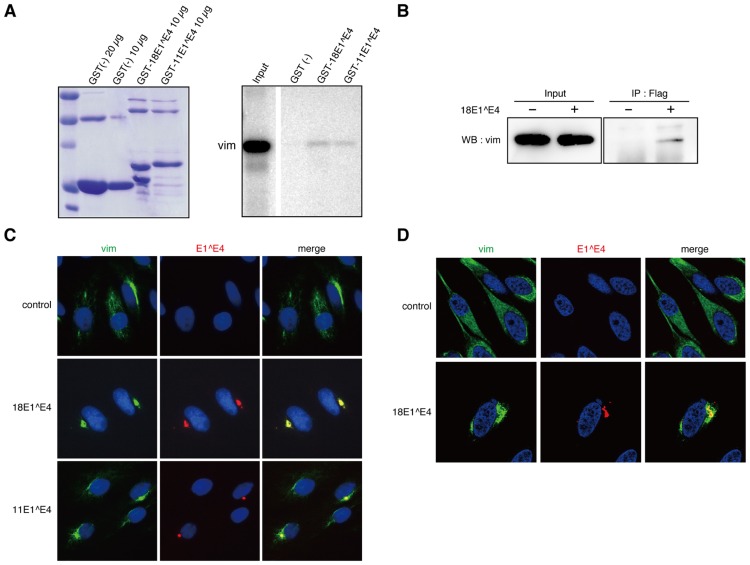
**Interaction between 18E1^E4 and vimentin. (A)** GST-pull down experiment was performed with GST-E1^E4 and *in vitro* translated vimentin. The GST-fusion proteins used for the experiment were checked by Coomassie Brilliant Blue staining (left panel). *In vitro* translated vimentin bound to GST protein (10 μg) was shown in right panel. “Input” indicates the *in vitro* translated vimentin used for each binding assay. **(B)** Binding of ectopically expressed FLAG-18E1^E4 to endogenous vimentin. 293T cells were transfected with FLAG-18E1^E4 expression plasmid, and the endogenous vimentin bound to FLAG-18E1^E4 was detected by immunoprecipitation with anti-FLAG antibody followed by immunoblot with anti-vimentin antibody. **(C)** Co-localization of endogenous vimentin (vim, green) and FLAG-18E1^E4 (E1^E4, red) in CV1 cells. Nuclei were stained with DAPI. Note that the co-localization could be observed in majority of the cells (≥70%) in that FLAG-18E1^E4 aggregates were detected. **(D)** Fine analysis of intracellular localization with confocal microscopic analysis. FLAG-18E1^E4 aggregate (red) was wrapped with vimentin (vim, green) in CV1 cells. Control shows mock transfected cells.

The interaction between 18E1^E4 and vimentin was confirmed by the *in vitro* binding assay (**Figure 1A**). We could detect weak but significant interaction between GST-tagged 18E1^E4 and vimentin obtained by *in vitro* translation, indicating the direct binding of 18E1^E4 to vimentin. Similar binding activity was also detected between HPV11 E1^E4 (11E1^E4) and vimentin (**Figure 1A**).

Next, we examined the interaction between endogenous vimentin and ectopically expressed 18E1^E4 in 293T cells. For the experiment, a FLAG epitope-tag was added at the N-terminus of 18E1^E4. The FLAG-18E1^E4 was immunoprecipitated with anti-FLAG antibody, and then co-precipitated vimentin was detected by immunoblotting analysis. As shown in **Figure 1B**, 18E1^E4 could interact with endogenous vimentin.

Intracellular localizations of 18E1^E4 and vimentin were analyzed with CV1 cells, monkey kidney epithelial cells negative for papillomavirus infection. In control cells, vimentin showed filamentous distribution throughout the cytoplasm (**Figure 1C**). The ectopically expressed 18E1^E4 formed aggregates in cytoplasm, as reported previously (**Figure 1C**; Nakahara et al., 2002). In 18E1^E4-expressing cells, vimentin was co-localized at the E1^E4 aggregates. 11E1^E4 could also form aggregates with vimentin (**Figure 1C**). The fine localization of 18E1^E4 and vimentin was examined with confocal microscopic analysis, and it was found that the aggregate was wrapped by vimentin (**Figure 1D**). These results indicated that 18E1^E4 and vimentin were associated *in vivo*, and suggested that 18E1^E4 recruited vimentin to its aggregates through this interaction.

### E1^E4 WAS ASSEMBLED INTO AGGRESOME-LIKE COMPARTMENT

It is known that cytoplasmic aggregates are organized in cells infected with several viruses; the aggregate is called an “aggresome” (Wileman, 2007). Aggresomes are structures assembled close to the MTOC. They contain molecular chaperones, ubiquitinated proteins, proteasomes, and HDAC6, and are wrapped with a vimentin cage (Rodriguez-Gonzalez et al., 2008). 18E1^E4 formed aggregates on the periphery of a nucleus and was associated with vimentin as shown in **Figures 1C, D**, raising the possibility that the E1^E4 proteins were assembled in an aggresome-like compartment.

**FIGURE 2:**
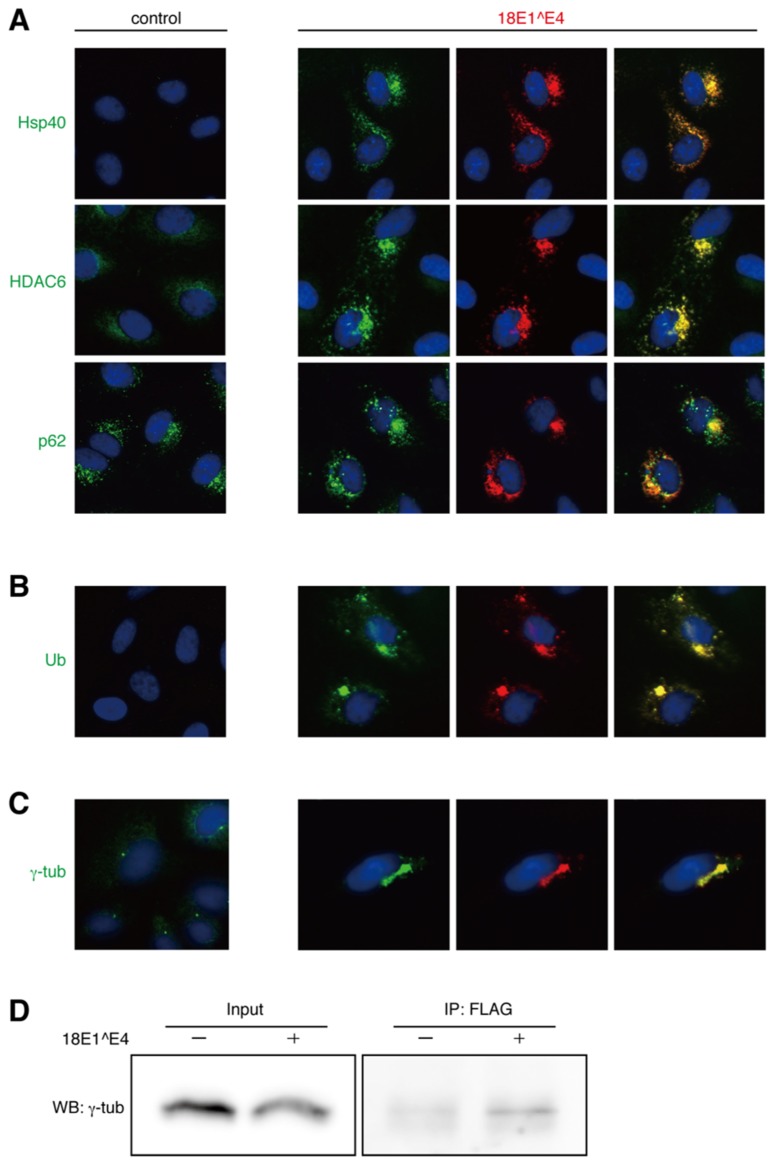
**Intracellular localization of “aggresome”-associated factors. (A)** Co-localization of endogenous Hsp40, HDAC6, or p62 (green) with FLAG-18E1^E4 (red) in CV1 cells. Nuclei were stained with DAPI. Control shows mock transfected cells. **(B)** Ubiquitinated proteins (Ub, green) were accumulated in FLAG-18E1^E4 aggregates (red) in CV1 cells. **(C)** γ-tubulin was localized at centrosomes in control cells (control). In FLAG-18E1^E4 expressing CV1 cells, γ-tubulin (γ-tub, green) was co-localized at the E1^E4 (red). For **(A–C)**, the co-localizations could be observed in most of the cells (>90%) in that 18E1^E4 aggregates were detected. **(D)** The cell lysates were obtained from the 293T cells transfected with FLAG-18E1^E4 expression plasmid. The lysates were used for immunoprecipitation with anti-FLAG antibody, and then the precipitates were analyzed by immunoblot with anti-γ-tubulin antibody.

We examined the intracellular localizations of 40 kDa heat shock protein (Hsp40), HDAC6, and p62, all of which were known to be assembled in the aggresome (Johnston, 2006). From immunofluorescence analysis of CV1 cells, it appeared that these factors were co-localized with the 18E1^E4-containing aggregates (**Figure 2A**). Because ubiquitinated proteins have been known to be recruited to the aggresome (Johnston, 2006), their localizations were also analyzed using anti-ubiquitin antibody. As shown in **Figure 2B**, ubiquitinated proteins were accumulated in the 18E1^E4 aggregates. These observations indicated that the 18E1^E4 aggregate had an aggresome-like composition. These results suggested that 18E1^E4 formed an aggresome-like compartment, called “18E1^E4-aggresome” hereafter.

It is considered that aggresomes are assembled by recruiting their components by retrograde transport through microtubules and are located close to MTOC. We analyzed the localization of γ-tubulin, a component of MTOC (**Figure 2C**). In control cells, γ-tubulin appeared at the centrosome as small dots in the perinuclear region. In the cells expressing 18E1^E4, γ-tubulin was co-localized at the 18E1^E4-aggresome, and the normal centrosome could not be detected in those cells, suggesting that 18E1^E4-aggresome formation disrupted the normal centrosome or MTOC structure.

The finding that γ-tubulin was co-localized at the 18E1^E4-aggresome urged us to investigate the interaction between γ-tubulin and 18E1^E4. 18E1^E4 with a FLAG-epitope tag at its N-terminus was expressed in 293T cells, and anti-FLAG antibody was used for immunoprecipitation of 18E1^E4-containing complexes. The complexes were analyzed by immunoblot detection with anti-γ-tubulin (**Figure 2D**). The result indicated the interaction between 18E1^E4 and γ-tubulin, which might be involved in the co-localization of γ-tubulin at the 18E1^E4-aggresome as observed in **Figure 2C**.

### DYNEIN-DEPENDENT FORMATION OF 18E1^E4 AGGRESOME

Misfolded/ubiquitinated proteins are connected to dynein, a motor protein, the association of which is mediated by HDAC6 as a linker molecule (Johnston, 2006). This complex is transported along microtubule filaments to the proximate region of MTOC and forms an aggresome (Kawaguchi et al., 2003). Nocodazole treatment interferes with the polymerization of microtubules and prevents aggresome formation.

**FIGURE 3:**
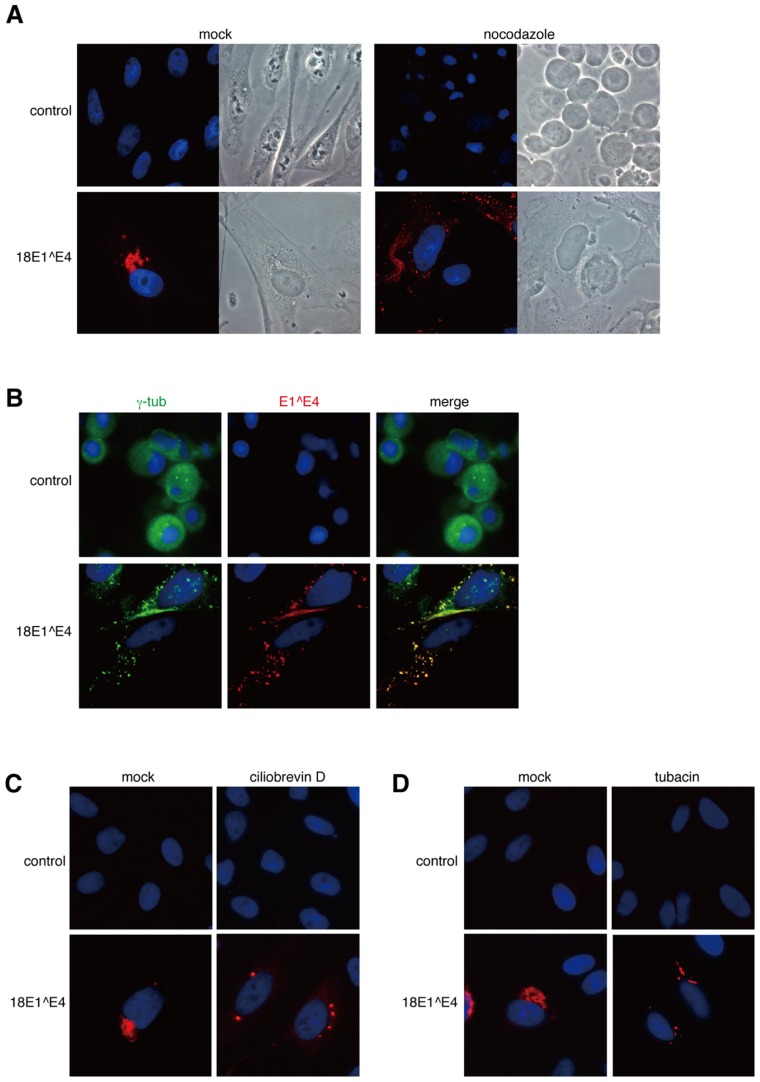
**Dynein-dependent transport through microtubule filaments was required for the assembly of 18E1^E4-aggresome. (A)** Nocodazole treatment disrupted the 18E1^E4-aggresome assembly. HeLa cells were transfected with FLAG-18E1^E4 expression plasmids, treated by nocodazole (10 mM) at 24 h after transfection. At 24 h after the treatment, the cells were fixed by 4% PFA, then stained with anti-FLAG antibody (red). Control shows untransfected cells, and “mock” indicates mock-treated cells. **(B)** γ-tubulin (green) was associated with small aggregates of FLAG-18E1^E4 (red) in the nocodazole-treated cells. The association could be detected in most of the cells (≥90%) that were positive for FLAG-18E1^E4 expression. Cells were prepared and treated as in **(A)**, except for fixation by cold methanol. **(C**, **D)** Ciliobrevin D (a dynein inhibitor) and tubacin (an HDAC6 inhibitor) treatments prevented 18E1^E4-aggresome assembly. Cells were prepared, fixed, and stained as in **(A)**, except for the inhibitors.

Nocodazole treatment of normal HeLa cells induced early M-phase cell cycle arrest and the cells were round (control, **Figure 3A**). In contrast, 18E1^E4-expressing cells were flat (18E1^E4, **Figure 3A**). We reported that 18E^E4 expression induced G2/M cell cycle arrest and accumulation of aneuploid cells (≥4N; Nakahara et al., 2002), suggesting that the cells were maintained in S and G2 phases of the cell cycle. By nocodazole treatment, the formation of 18E1^E4-aggresome was significantly inhibited and small aggregates of 18E1^E4 were broadly distributed in the cytoplasm, indicating that the assembly of 18E1^E4-aggresome required functional microtubule networks. We could detect γ-tubulin in 18E1^E4 small aggregates in nocodazole-treated cells (**Figure 3B**), suggesting that 18E1^E4 associated with γ-tubulin in cytoplasm and assembled it to an 18E1^E4-aggresome in a microtubule-dependent manner.

A similar experiment was performed with a dynein inhibitor, ciliobrevin D (**Figure 3C**). Ciliobrevin D treatment strongly suppressed E1^E4-aggresome formation, indicating that dynein-dependent transport was involved in E1^E4-aggresome formation.

The effect of an HDAC6 inhibitor, tubacin, was also tested (**Figure 3D**). HDAC6 is important for aggresome formation by loading the cargo containing misfolded/ubiquitinated proteins onto a dynein motor (Kawaguchi et al., 2003). Tubacin treatment disrupted the E1^E4-aggresome and small aggregates containing 18E1^E4 were detected in the cytoplasm, as in the cases of nocodazole and ciliobrevin D treatments.

These results suggested that the 18E1^E4-aggresome was assembled by dynein-dependent retrograde transport along microtubule filaments.

### PROTEASOME INHIBITOR AUGMENTED E1^E4-AGGRESOME FORMATION

In the cytoplasmic region, proteasomes are located around the centrosome, close to cytoskeletal networks and on the surface of the endoplasmic reticulum (ER), and the centrosome region is considered as the major site for proteasome-dependent proteolysis, called the proteolysis center (Wójcik and DeMartino, 2003). It was reported that inhibition of proteasome function accelerated aggresome formation in the centrosome region (Johnston et al., 1998), which is considered as one of the hallmarks of aggresomes.

**FIGURE 4:**
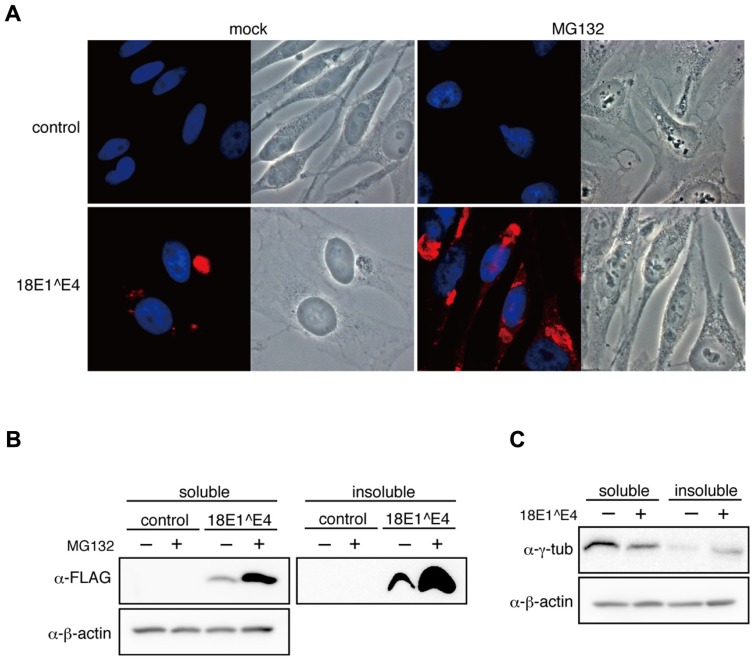
**Effect of proteasome inhibitor on 18E1^E4-aggresome assembly. (A)** MG132 treatment augmented the aggresome assembly. HeLa cells were transfected with FLAG-18E1^E4 expression plasmid, treated with MG132 (10 mM) at 24 h after transfection. “mock” indicates mock-transfected cells. Cells were fixed by 4% PFA at 24 h after treatment, then stained with anti-FLAG antibody (red). Control shows mock transfected cells. **(B)** Cells prepared as shown in **(A)** were used to obtain cell lysates. The soluble and insoluble fractions were immunoblotted with anti-FLAG antibody. Most part of 18E1^E4 protein was found in the insoluble fraction of cell lysate. MG132 treatment increased the amounts of 18E1^E4 in both fractions. **(C)** Immunoblot analysis was performed as shown in **(B)** with anti-γ-tubulin antibody. γ-tubulin was partially sequestrated in the insoluble fraction by 18E1^E4.

We examined the effect of MG132, a proteasome inhibitor, on cells expressing 18E1^E4, and found that MG132 treatment augmented 18E1^E4-aggresome formation (**Figure 4A**). This observation was consistent with the idea that 18E1^E4 formed aggresome-like compartment.

The expression levels of 18E1^E4 were examined in MG132-treated cells. As reported previously (Nakahara et al., 2002), most of 18E1^E4 was found in the insoluble fraction of cell lysate (**Figure 4B**), which was corresponding to 18E1^E4-aggresome formation. With MG132, 18E1^E4 in the insoluble fraction was increased significantly, reflecting the augmentation of aggresome formation. Surprisingly, 18E1^E4 in the soluble fraction was also increased, suggesting that some portion of 18E1^E4 was processed in proteasome-dependent manner (**Figure 4B**).

18E1^E4 proteins were assembled into aggresomes as insoluble fraction of cell lysate, indicating that the factors recruited to 18E1^E4-aggresomes might be sequestrated as insoluble materials. As shown in **Figures 2C, D**, γ-tubulin was associated with 18E1^E4 and recruited to the aggresomes. We examined the effect of 18E1^E4 expression on the protein levels of γ-tubulin (**Figure 4C**). The amounts of soluble γ-tubulin were reduced by 18E1^E4 expression. On the contrary, those in the insoluble fraction were increased, suggesting that γ-tubulin was sequestrated into the 18E1^E4-aggresome as insoluble material, which might reduce active fraction of γ-tubulin and disturb normal centrosome/MTOC formation as shown in **Figure 2C**.

### 18E1^E4 AGGRESOME WAS INVOLVED IN THE TURN OVER OF HPV ONCOPROTEINS

As described above, 18E1^E4 could sequestrate γ-tubulin in the aggresome. In considering the involvement of 18E1^E4-aggresome in HPV replication, we examined the possibility that the aggresome contributed to sequestration of other viral proteins.

**FIGURE 5:**
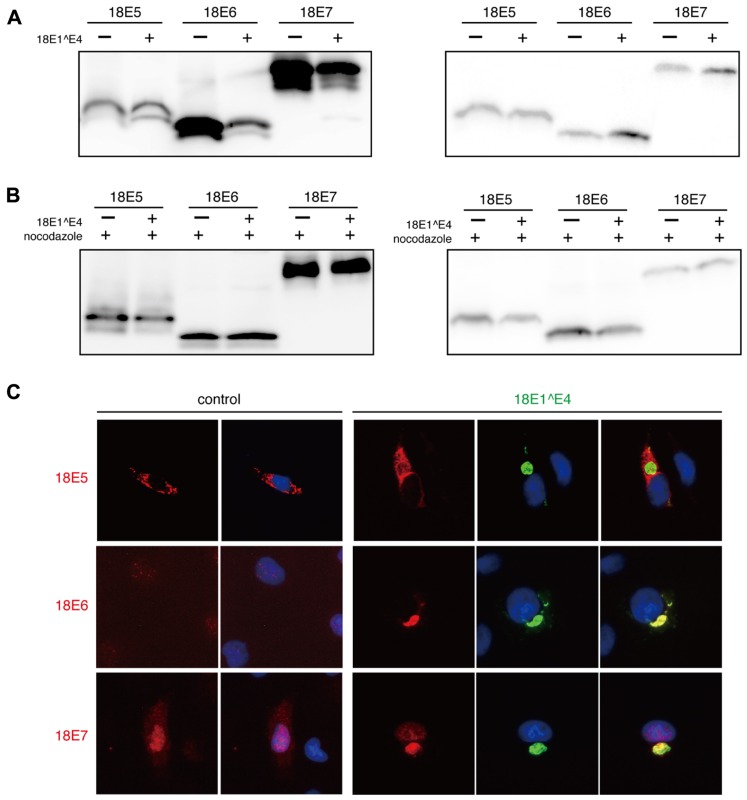
**Major viral oncoproteins were sequestrated in 18E1^E4-aggresome. (A)** CV1 cells were co-transfected with 18E1^E4 and FLAG-tagged 18E5, 18E6, or 18E7 expression plasmids. At 48 h after transfection, cells were lysed by triple detergent buffer. The expression levels of HPV18 E5, E6, and E7 were analyzed both in soluble (left panel) and insoluble fractions (right panel) of cell lysates. **(B)** The effect of nocodazole treatment (10 mM) was examined by a similar experiment as shown in **(A)**. **(C)** Intracellular localization of FLAG-tagged 18E5, 18E6, or 18E7 (red) with EGFP-tagged 18E1^E4 (green) in CV1 cells. Nuclei were stained with DAPI. The colocalization could be detected in most of the cells (≥90%) that were positive for EGFP-tagged 18E1^E4 expression.

In CV1 cells, FLAG-epitope tagged 18E5, 18E6, or 18E7 was expressed with or without 18E1^E4, and then the expression level was monitored by immunoblotting analysis (**Figure 5A**). Although the expression of E5 was not affected, those of E6 and E7 in the soluble fraction were significantly reduced by 18E1^E4 and accumulation of those proteins in insoluble material was observed. This result suggested that E6 and E7 were sequestrated in 18E1^E4-aggresomes. Nocodazole treatment blocked the effect of 18E1^E4 (**Figure 5B**), suggesting that the 18E1^E4-aggresome formation was involved in sequestration of 18E6 and18E7.

In the cells expressing 18E1^E4, E6 and E7 were co-localized at 18E1^E4-aggresomes (**Figure 5C**). The localization of 18E5 was not altered by 18E1^E4 expression. These observations indicated that major viral oncoproteins, E6 and E7, were recruited to the 18E1^E4-aggresome and sequestrated in insoluble materials.

## DISCUSSION

It was reported that ectopically expressed HPV E1^E4 formed aggregates in cytoplasm (Doorbar et al., 1991), although the function of the aggregate remained to be clarified. In this paper, we described that 18E1^E4 was assembled into an aggresome-like compartment (18E1^E4-aggresome) and was involved in the sequestration of viral oncoproteins.

### AGGRESOME-LIKE COMPARTMENT FORMATION BY 18E1^E4.

We found that 18E1^E4 interacted with vimentin and recruited it to the 18E1^E4 aggregates (**Figure 1**), which inspired us to consider that 18E1^E4 was assembled into an aggresome-like compartment because aggresomes are known to be wrapped by vimentin.

There is a report that 16E1^E4 could interact with cytokeratins 8/18 (CK8/18) but not with vimentin (Wang et al., 2004). We therefore analyzed the interaction between 18E1^E4 and endogenous vimentin both *in vivo* and *in vitro* (**Figures 1B, C**), although they used an *in vitro* binding assay with recombinant vimentin and 16E1^E4. The different experimental condition could be the cause of the controversial observations.

Aggresomes are assembled to process misfolded/ubiquitinated proteins that are not well handled by the ubiquitin-proteasome pathway or the chaperone-dependent refolding system (Goldberg, 2003). It is known that aggresomes incorporate molecular chaperones, ubiquitinated proteins, p62 and HDAC6 (Rodriguez-Gonzalez et al., 2008). We confirmed that these molecules were recruited to the 18E1^E4-aggregate (**Figures 2A,B**). This observation strongly suggested that 18E1^E4 aggregate had an aggresome-like structure.

Aggresome formation is dependent on microtubules and dynein. Microaggregates of misfolded proteins are transported to MTOC along microtubules in a dynein-dependent manner (Johnston, 2006). Dynein is a motor protein and microaggregates are linked to dynein through HDAC6 (Kawaguchi et al., 2003). We examined the effects of nocodazole, an inhibitor of microtubule polymerization; ciliobrevin D, a dynein inhibitor; and tubacin, a HDAC6 inhibitor, on 18E1^E4 aggregate formation, and found that all of the inhibitors could efficiently interfere with aggregate formation (**Figure 3**). This result supported the possibility that 18E1^E4 was assembled in the aggresome-like compartment, 18E1^E4-aggresome. We are currently investigating a role of the interaction between 18E1^E4 and vimentin in aggresome formation.

### DISRUPTION OF MTOC BY 18E1^E4

Aggresomes are known to be assembled close to MTOC (Johnston et al., 1998). We examined the localization of γ-tubulin, a component of MTOC, in 18E1^E4-expressing cells, and found that it was co-localized at the 18E1^E4-aggresome (**Figure 2C**). Direct interaction was found between 18E1^E4 and γ-tubulin (**Figure 2D**), by which γ-tubulin might be recruited to the E1^E4-aggresome. Even though nocodazole treatment inhibited E1^E4-aggresome formation, colocalization of 18E1^E4 and γ-tubulin could be detected (**Figure 3B**). It was also found that regular centrosome or MTOC formation was disrupted in 18E1^E4 expressing cells (**Figure 2C**). Proper assembly of MTOC is essential for mitotic events (Bettencourt-Dias and Glover, 2007), and the disturbance of MTOC formation by 18E1^E4 might contribute to the G2/M cell cycle arrest induced by 18E1^E4.

### POSSIBLE ROLE OF 18E1^E4 AGGRESOME IN VIRUS REPLICATION

Although it is known that aggresome formation has a protective role against bacterial and protozoal infections (Wileman, 2007), several viruses are reported to utilize aggresomes for their replication processes (Wileman, 2007). Nucleocytoplasmic large DNA viruses (NCLDV), including poxviruses, African swine fever virus (ASFV), iridoviruses and phycodnaviruses, have been reported to utilize aggresomes as compartments for the accumulation of host and viral proteins, where virus replication and virion assembly are accelerated. It has been proposed that infection with a retrovirus or herpes virus produces an aggresome-like structure in the perinuclear region, which is utilized as a virus assembly site (Wileman, 2007). These findings suggested that the 18E1^E4-aggresome had a functional role in virus replication.

As shown in **Figures 2C, D**, 18E1^E4 bound to γ-tubulin and recruited it to aggresome-like compartment. This sequestration of γ-tubulin might cause disruption of normal centrosome/MTOC organization. We considered that the 18E1^E4-aggresome might be involved in sequestration of other viral proteins, especially of the viral oncoproteins. We examined the effect of 18E1^E4 on the expression levels of 18E5, 18E6, and 18E7 (**Figure 5A**). Although the expression level of E5 did not altered by 18E1^E4, those of E6 and E7 in soluble fraction were severely reduced. E6 and E7 were found in insoluble fraction and co-localized at 18E1^E4-aggresomes (**Figures 5A, C**). These observations suggested that 18E1^E4 sequestrated E6 and E7 into the inactive aggregate and reduced active fractions of them. We could not detect direct binding activity of 18E1^E4 to 18E6 or 18E7 (data not shown), and therefore it will be necessary to clarify the mechanism by which E6 and E7 are recruited to the aggresome.

Most 18E6 and 18E7 are partitioned in soluble fraction as shown in **Figure 5A**. The amounts of these proteins in soluble fraction were significantly reduced by 18E1^E4 expression, although those in insoluble fraction were increased modestly. The result suggested that 18E1^E4 expression reduced the total amounts of these oncoproteins in the cells possibly by accelerating their turn over. We are now investigating the effect of 18E1^E4 expression on total amounts of the viral oncoproteins.

In lesions infected with cutaneous-type HPVs, HPV1, HPV4, and HPV63, E1^E4 aggregate could be detected in upper layers of the warts as intracytoplasmic inclusion bodies (Egawa, 1994). In the case of HPV16 infection, it was reported that inclusion bodies of E1^E4 were found in differentiated layers of cervical intraepithelial neoplasia grade 1 (CIN1) lesions (Doorbar et al., 1997; Doorbar, 2005). These observations suggest that the E1^E4-aggresome functions in the upper layers of the infected lesion.

Here we propose a model of E1^E4 function in viral replication. In basal and parabasal cells of HPV-infected lesions, viral oncoproteins, E6 and E7, are expressed from the viral early promoter. This suppresses cell differentiation and promotes cell proliferation (Nguyen et al., 2003; Ueno et al., 2006), which is required for expanding the population of infected cells. As cellular differentiation progresses, the viral late promoter is activated and directs the expression of E1^E4. E1^E4 causes G2/M cell cycle arrest and activates endoreduplication (Nakahara et al., 2005). This cellular condition favors genome amplification and gene expression of the virus. Then the high-level expression of E1^E4 induces aggregate formation in upper layers of the lesion, where the E1^E4-aggresome sequestrates E6 and E7, suppresses their inhibitory effect on cellular differentiation and induces terminal differentiation. Terminal differentiation is required for capsid protein expression and virion assembly, although the underlying mechanism remains unknown (Sakakibara et al., 2013).

It was reported that the formation of E1^E4 aggregates disrupted cytokeratin networks and might be helpful for virion egress from keratinized cells (Doorbar et al., 1991). This idea is very attractive for an E1^E4 function, and it is important to verify these E1^E4 functions in an animal infection model, histological analysis of human samples, or an organotypic raft culture system.

## Conflict of Interest Statement

The authors declare that the research was conducted in the absence of any commercial or financial relationships that could be construed as a potential conflict of interest.
